# Circulating CD14^+^HLA-DR^−/low^ Myeloid-Derived Suppressor Cells as Potential Biomarkers for the Identification of Psoriasis TCM Blood-Heat Syndrome and Blood-Stasis Syndrome

**DOI:** 10.1155/2020/4582459

**Published:** 2020-04-14

**Authors:** Shipeng Sun, Yali Wei, Xue Zeng, Yuliang Yuan, Na Wang, Cheng An, Jinlong Duan, Bo Pang, Zifu Hong, Guijian Liu

**Affiliations:** ^1^Clinical Laboratories, Guang'anmen Hospital, China Academy of Chinese Medical Sciences, Beijing, China; ^2^Department of Dermatology, Guang'anmen Hospital, China Academy of Chinese Medical Sciences, Beijing, China; ^3^South Area of Guang'anmen Hospital, China Academy of Chinese Medical Sciences, Beijing, China; ^4^Department of Proctology, Guang'anmen Hospital, China Academy of Chinese Medical Sciences, Beijing, China

## Abstract

Psoriasis is a chronic autoimmune disease. Identification of the biomarkers responsible for Traditional Chinese Medicine (TCM) syndromes of psoriasis can help researchers recognize the different aspects of psoriasis and find novel therapeutic targets for the treatment of psoriasis. The current study investigated the levels of circulating Mo-MDSCs and Mo-MDSC-associated immune factors in the peripheral blood of psoriasis patients with different TCM syndromes. We found that the frequency of Mo-MDSCs (CD14^+^HLA-DR^−/low^ cells) among CD14^+^ cells from plaque psoriasis patients with blood-stasis (BS) syndrome was significantly increased when compared with healthy controls (*p* < 0.001) and blood-heat (BH) syndrome group (*p* < 0.001), respectively. However, serum IL-2, IL-4, IL-6, IL-10, IL-17A, TNF-*α*, IFN-*γ*, iNOS, Arg-1, and NO concentration showed no statistically significant difference between healthy controls and psoriasis patients as well as no significant difference between the BH and BS syndrome groups. Compared with healthy controls, the mRNA expression of Arg-1, TNF-*α*, ROR-*γ*, and PD-L1 was increased, while the mRNA expression of PD-1 and IL-10 was decreased in PBMCs from psoriasis patients. Moreover, the mRNA expression of TNF-*α* and FOXP3 in PBMCs showed a pronounced statistical difference between the psoriatic BH syndrome group and the BS syndrome group. Therefore, we provide evidence that the percentage of CD14^+^HLA-DR^−/low^ MDSC/ CD14^+^ cells and TNF-*α* and Foxp3 mRNA expression levels in PBMCs are potential biomarkers for distinguishing TCM BH syndrome and BS syndrome.

## 1. Introduction

Psoriasis is a chronic autoimmune disease, which mostly affects the skin [1]. Classical psoriasis is a T-cell mediated autoimmune disease that is primarily driven by autoreactive T cells that produce high levels of interleukin-17 (IL-17) in response to IL-23 and tumor necrosis factor-alpha (TNF-*α*) [2]. There is no curative treatment, but symptoms can be relieved through various treatment strategies including topical, systemic, phototherapy, and biologicals [3]. The long-term use of immunosuppressive agents within these approaches leads to immunological side effects and might increase the risk of infections [3, 4]. Traditional Chinese Medicine (TCM) is one of the oldest forms of traditional medicine with a history of over 3000 years [5]. Clinical and experimental data indicate that TCM could treat psoriasis by antagonizing or regulating the IL-23/IL-17 axis leading to inhibition of the main pathophysiological pathways [6, 7].

In clinical practice, practitioners form diagnoses and prepare prescriptions mainly based on TCM syndromes (also called “ZHENG”), which are identified through medical history, symptoms, and signs that vary between individuals [5, 8]. The most common form of psoriasis is plaque psoriasis, and it is present in roughly 90% of psoriasis patients. Psoriasis can be classified into three clinical stages with regard to disease activity. During the acute clinical stage of psoriasis, lesions are flared, unstable, and sometimes accompanied by Köbner's phenomenon. This acute stage is also referred to as blood-heat (BH) syndrome. At the stationary clinical stage of psoriasis, lesions are stable and indelible, but no fresh lesions appear. The stationary clinical stage is known as TCM blood-stasis (BS) syndrome [7]. Different TCM syndromes might imply relatively distinct pathogeneses within the same disease. Identification of the biomarkers associated with TCM syndromes of psoriasis may help researchers understand the different aspects of psoriasis better and find novel therapeutic targets. However, the study of the correlations between TCM syndromes and laboratory data has been limited so far. In order to reveal the distinct mechanisms in psoriatic TCM BH syndrome and BS syndrome, potential biomarkers for the identification of these two psoriatic TCM syndromes should be explored by analyzing and summarizing relevant laboratory data.

Myeloid-derived suppressor cells (MDSCs), a heterogeneous group of immature myeloid cells with highly immunosuppressive activity [9], are divided into three cell types: granulocytic or polymorphonuclear MDSCs (G-MDSCs/PMN-MDSCs), monocytic MDSCs (Mo-MDSCs), and early-stage MDSC (E-MDSC) [10]. MDSC expansion and activation are mainly driven by various inflammatory factors, such as granulocyte-macrophage colony-stimulating factor, TNF-*α*, IL-1*β*, IL-6, interferon-*γ* (IFN-*γ*), transforming growth factor-*β* (TGF-*β*), and vascular endothelial growth factor (VEGF) [11]. The hallmark of MDSCs is their ability to suppress T-cell and NK cell responses, while inducing a state of tolerance. Different subsets of MDSCs adopt distinct specific strategies for exerting their immunosuppressive effect. PMN-MDSCs mainly suppress antigen-specific T-cell responses by producing reactive oxygen species (ROS). M-MDSCs produce high amounts of NO, Arginase I (Arg-1), and immunosuppressive cytokines, such as IL-10, which suppress both antigen-specific and nonspecific T-cell responses. Mo-MDSCs are more potent suppressors than PMN-MDSCs because of NO, which has a longer half-life than ROS and reacts at greater distances [12]. A study revealed that Mo-MDSC (CD14^+^HLA-DR^−/low^) fractions were elevated in psoriasis patient peripheral blood mononuclear cells (PBMCs) compared to healthy controls, and, importantly, regulatory T cells induced by psoriasis Mo-MDSCs displayed decreased suppressive functionality [13]. Nevertheless, the pathophysiological mechanism and immunosuppressive functions of MDSCs in different TCM syndromes of psoriasis are poorly understood. The aim of the present study was to identify potential biomarkers for the identification of psoriasis TCM BH syndrome and BS syndrome by investigating circulating Mo-MDSCs and Mo-MDSC-associated immune factors in the peripheral blood of psoriasis patients with different TCM syndromes.

## 2. Materials and Methods

### 2.1. Study Participants

Study participants included the following: 20 healthy control subjects without inflammatory skin disease and 47 patients with plaque psoriasis, including 24 psoriatic BS syndrome patients and 23 psoriatic BH syndrome patients. Blood samples were collected from all study participants between May 2018 and September 2019. All participants had given their written informed consent to institutional protocols approved by the Guang'anmen Hospital, China Academy of Chinese Medical Sciences Ethics Committee (reference no. 2018-007-KY-02). Inclusion criteria included the following: (1) psoriasis patients or healthy control subjects older than 18 years of age, (2) patients able to give written informed consent, and (3) patients able to give blood samples. Exclusion criteria covered the following: (1) psoriasis patients or healthy control subjects less than 18 years of age; (2) patients having other types of psoriasis such as guttate psoriasis, inverse psoriasis, pustular psoriasis, erythrodermic psoriasis, and arthropathic psoriasis; (3) patients having used topical medications (including Vitamin D3 analogues, retinoids, and corticosteroids) within the past 2 weeks; (4) patients having used systemic medications (including corticosteroids, biologic agents, acitretin, cyclosporine, and methotrexate) within the past 4 weeks; (5) patients concomitant with pregnancy, tumors, infections, and hematological diseases; and (6) rejection to give written informed consent or blood samples. All procedures performed in experiments involving human participants were carried out in accordance with the ethical standards of the institutional and/or national research committee and the 1964 Helsinki declaration and its later amendments or comparable ethical standards.

### 2.2. Diagnostic Criteria

Diagnostic criteria of TCM psoriatic BH and BS syndrome were based on the Guiding Principles for Evidence-based Clinical Practice of Traditional Chinese Medicine in Psoriasis Vulgaris (2013 Edition) [14]. Primary symptoms include bright red skin lesions and the continuous appearance of new rashes (mainly maculopapular). Accompanying symptoms include easily to be upset and irritable; yellow urine; red or crimson tongue; and wiry, rolling, rapid pulse. Participants with all the primary symptoms and any of the accompanying symptoms would be diagnosed as having psoriasis with BH syndrome.

Primary symptoms include dark red skin lesions, hypertrophic infiltration, and prolonged recovery from skin lesions. Accompanying symptoms include scaly dry skin, blackish complexion or cyanosis of the lips and nails, dark menstruation or with blood clots, dark purple color and petechial bruise, and astringent pulse or thin and moderate pulse. Participants with all the primary symptoms and any one of the accompanying symptoms would be diagnosed as having psoriasis with BS syndrome.

### 2.3. Flow Cytometry Analysis

Five milliliters of venous peripheral blood from patients and healthy volunteers was collected in EDTA-coated tubes. Samples of 50 *μ*L blood per tube were transferred for staining with monoclonal antibodies and red blood cell (RBC) lysis. Peripheral blood samples were stained according to the manufacturer's recommendations using the following fluorochrome-coupled antibodies: anti-CD14-FITC (IM0645U, Beckman Coulter Company), anti-HLA-DR-PB (A74781, Beckman Coulter Company), and anti-CD45-KO (A96416, Beckman Coulter Company). Whole blood cells were incubated with antibodies against surface markers at room temperature (approximate range 20°C to 25°C) for 15 min in the dark. After RBC lysis washing, cells were collected and analyzed with Navios Cytometer (Beckman Coulter Company). Mo-MDSCs were defined as CD45^+^, CD14^+^, and HLA-DR^low/−^. Isotype-matched antibodies were used in all samples as controls. Specifically, the population defined as HLA-DR^low/−^ was based on isotype staining with gating to include, at a minimum, 95% of the CD14^+^ population.

### 2.4. Determination of Serum Immune Factors

IL-2, IL-4, IL-6, IL-10, IL-17A, TNF-*α*, and IFN-*γ* were quantified in sera from healthy controls and subjects with psoriasis by Th1/Th2/Th17 cytokine assay (JiangXi Cellgene Biotech Co., LTD, China) according to the manufacturers' instructions. Data were acquired using a Navios Cytometer (Beckman Coulter Company). Standard curves were constructed, and calculations were performed using JiangXi Cellgene Biotech Co., LTD CBA software.

Arg-1 was quantified in sera from healthy controls and subjects with psoriasis by a quantitative colorimetric arginase determination assay (Quanti Chrom Arginase Assay Kit, DARG-200, Bioassay Systems) according to the manufacturer's instructions.

NO was quantified in sera from healthy controls and subjects with psoriasis using the NO kit (Moledia Technology Corp. of Beijing) and AU5822 (Beckman Coulter), according to the manufacturer's instructions. Serum iNOS level was quantified using iNOS Detection kits (A014-1, Nanjing Jiancheng Bioengineering Institute) according to the manufacturer's instructions.

### 2.5. Analysis of Mo-MDSC-Associated Immune Factor and Transcription Factor mRNA in PBMCs

Peripheral blood mononuclear cells (PBMCs) were obtained from EDTA-K2-treated venous blood by density gradient centrifugation using Human Lymphocyte Separation Medium (TIAN JIN HAO YANG BIOLOGICAL MANUFACTURE CO., LTD). RNA was extracted from PBMCs using the TRIzol kit (Thermo Fisher Scientific). cDNA was synthesized using PrimeScript™RT Reagent Kit (TAKARA) and qPCR was performed in triplicate using 10 mL of SYBR® Premix Ex Taq™ II (TAKARA). Primers used are listed in Table 1. All reactions included 40 cycles of 15 s at 95°C, followed by 1 min at 60°C. Relative gene expression was calculated using the 2^−ΔΔCT^ method and normalized to the corresponding level of glyceraldehyde 3-phosphate dehydrogenase (GAPDH).

### 2.6. Statistical Analysis

Data analysis was carried out retrospectively on prospectively collected samples. Patient characteristics were summarized as arithmetic means with SD or as medians with ranges, according to data distribution. Comparisons between groups were performed using the paired *t-*test or Mann–Whitney *U* test. Spearman's rank correlation analysis and linear regression analysis were performed to determine the association between variables. All tests were two-sided with a *p* ≤ 0.05 being considered as statistically significant. All data were analyzed using the SPSS software package version 20 and Prism v6.0 software (GraphPad Software, Inc).

## 3. Results

### 3.1. Demographics of the Study Cohort

Study participants included 20 healthy control subjects without inflammatory skin disease and 47 patients with psoriasis including 23 psoriasis patients with BH syndrome and 24 psoriasis patients with BS syndrome. Patient demographics are shown in Table 2. Blood samples were collected from all study participants, who had given their written informed consent to institutional protocols approved by the Guang'anmen Hospital, China Academy of Chinese Medical Sciences Ethics Committee (reference no. 2018-007-KY-02). Inclusion criteria included psoriasis patients or healthy control subjects older than 18 years of age, patients able to give written informed consent, and patients able to give blood samples. Exclusion criteria included patients on subcutaneous and intravenous systemic immunosuppressant medications.

### 3.2. Circulating Mo-MDSCs Are Increased in the Peripheral Blood of Patients with Psoriasis with Blood-Stasis Syndrome

**Figure 1:**
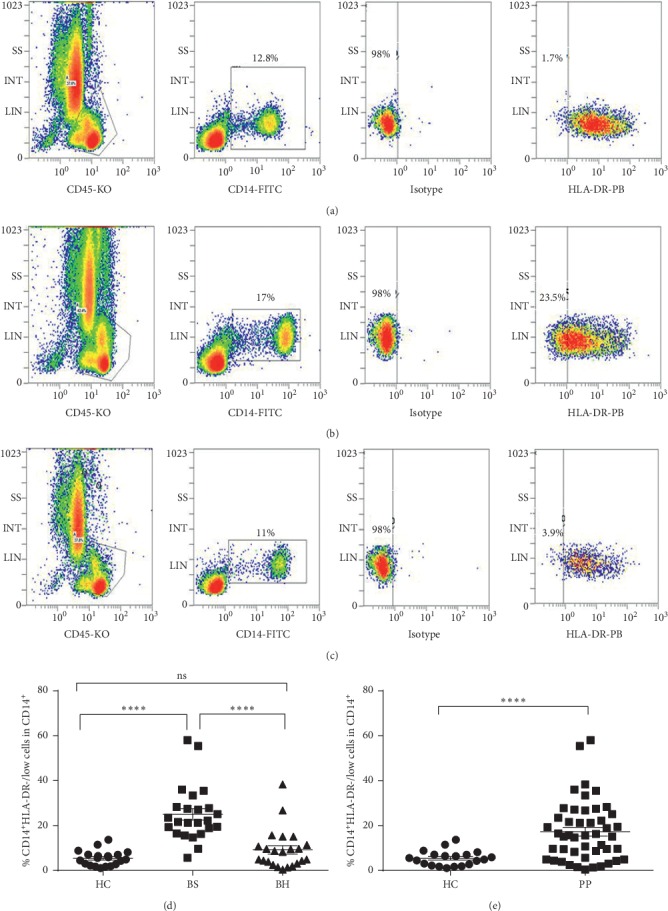
Frequency of circulating Mo-MDSCs is increased in the peripheral blood of patients with psoriasis with BS syndrome. Representative flow cytometry panels show quantification of Mo-MDSCs among PBMCs of healthy control subjects (a), psoriatic BS syndrome group (b), and psoriatic BH syndrome group (c). (d) The frequency of HLA-DR^−/low^ cells among CD14^+^ cells from psoriatic BS syndrome is significantly higher when compared to healthy controls or the psoriatic BH syndrome group, respectively (^*∗∗∗∗*^*p* < 0.0001). (e) The frequency of HLA-DR^−/low^ cells among CD14^+^ cells of psoriasis patients is higher when compared to healthy controls.

The frequency of HLA-DR^−/low^ cells among CD14^+^ cells of psoriasis patients with BS syndrome was significantly higher when compared with healthy controls (*p* < 0.001, Mann–Whitney nonparametric *U* test) and the BH syndrome group (*p* < 0.001, Mann–Whitney nonparametric *U* test). However, the frequency of HLA-DR^−/low^ cells among CD14^+^ cells showed no significant difference between psoriasis patients with BH syndrome and healthy controls (*p*=0.0648, Mann–Whitney nonparametric *U* test). Representative images demonstrating the fraction of Mo-MDSCs as a percentage of CD14^+^ cells from the blood of healthy controls or psoriasis patients are shown in Figure 1.

### 3.3. Linear Correlation of Circulating CD14^+^HLA-DR^−/low^ MDSCs Blood Levels with Psoriasis Severity

**Figure 2:**
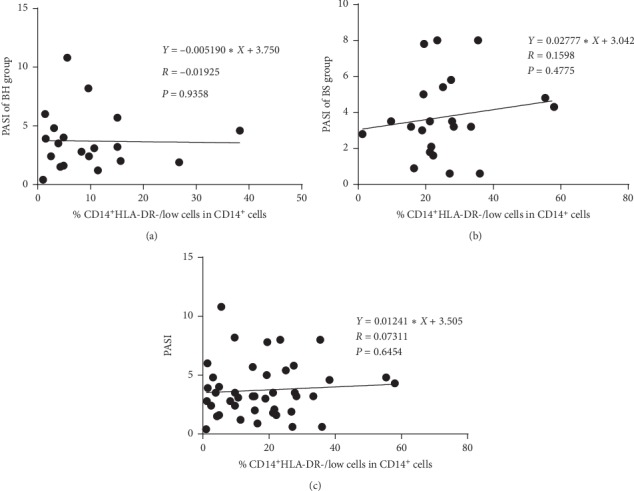
Correlation analyses of psoriasis severity as assessed by the psoriasis area and severity index (PASI) and the percentage of circulating Mo-MDSCs among CD14^+^ monocytes. (a) PASI and percentage of circulating Mo-MDSCs among CD14^+^ monocytes in the BH group (*n* = 20); (b) PASI and percentage of circulating Mo-MDSCs among CD14^+^ monocytes in the BS group (*n* = 22); (c) PASI and percentage of circulating Mo-MDSCs among CD14^+^ monocytes in all psoriasis patients.

Given that the fraction of CD14^+^HLA-DR^−/low^ MDSC among CD14^+^ cells positively correlated with psoriatic BS syndrome, we examined the relationship between the fraction of CD14^+^HLA-DR^−/low^ MDSC and disease severity, as assessed by the psoriasis area and severity index (PASI) score [15]. The results are shown in Figure 2. Statistical analysis of all patient data showed no correlation between the percentage of CD14^+^HLA-DR^−/low^MDSCs/ CD14^+^ cells and PASI scores in the BS group (*n* = 22, *r* = 0.1598, *p*=0.4775), BH group (*n* = 20, *r* = −0.019, *p*=0.9358), and all psoriasis patients (*n* *=* *4*2, *r* *=* 0.073, *p*=0.6454). Consistent with a previous study [16], the frequency of CD14^+^HLA-DR^−/low^ MDSC in blood was markedly increased in psoriasis patients when compared to healthy control subjects, yet there was no statistically significant relationship with disease severity (based on the PASI score).

### 3.4. Analysis of Serum Cytokines and Immunosuppressive Molecules

**Figure 3:**
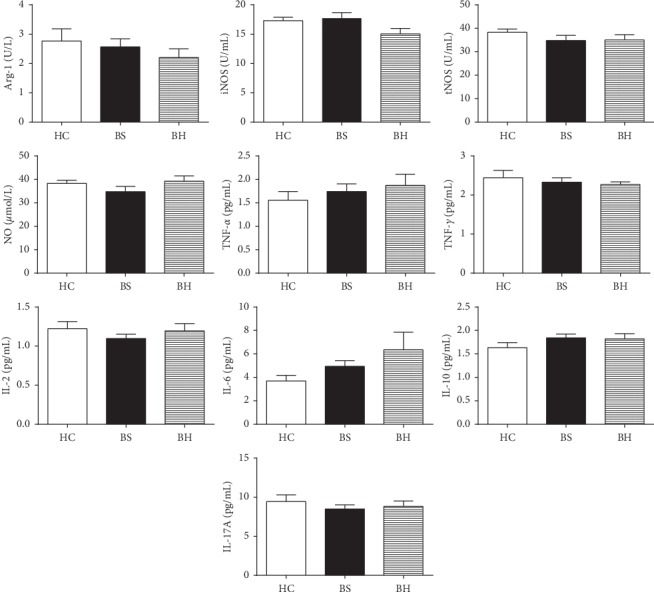
Detection of serum immune factor levels in psoriasis patients and healthy controls.

Proinflammatory cytokines, such as TNF-*α*, IL-6, and IL-1*β*, are drivers of MDSC expansion under pathological conditions. Mo-MDSCs exercise their immunosuppressive activities by the production of immunosuppressive factors such as IL-10, Arg-1, and iNOS to downregulate effector T-cell responses [17, 18]. To determine whether Mo-MDSC-associated immune factors are associated with different psoriasis TCM syndrome groups, we analyzed levels of serum iNOS, Arg-1, NO concentration, and cytokine levels (IL-2, IL-4, IL-6, IL-10, IL-17A, TNF-*α*, and IFN-*γ*) in healthy controls, the psoriatic BS syndrome group, and the psoriatic BH syndrome group. The results are presented in Figure 3. Levels of all immune factors showed no difference between groups (HC versus BH; HC versus BS; BS versus BH).

Values of sera IL-2, IL-4, IL-6, IL-10, IL-17A, TNF-*α*, IFN-γ, iNOS, Arg-1, and NO in psoriatic BS syndrome (*n* *=* 24) showed no significant difference (*p* > 0.05, compared to respective control by paired *t-*test or Mann–Whitney nonparametric *U* test) when compared with levels of healthy controls (*n* *=* 20) and the psoriatic BH syndrome group (*n* = 23), respectively.

### 3.5. Analysis of Mo-MDSC-Associated Immune Factor and Transcription Factor mRNA in PBMCs

**Figure 4:**
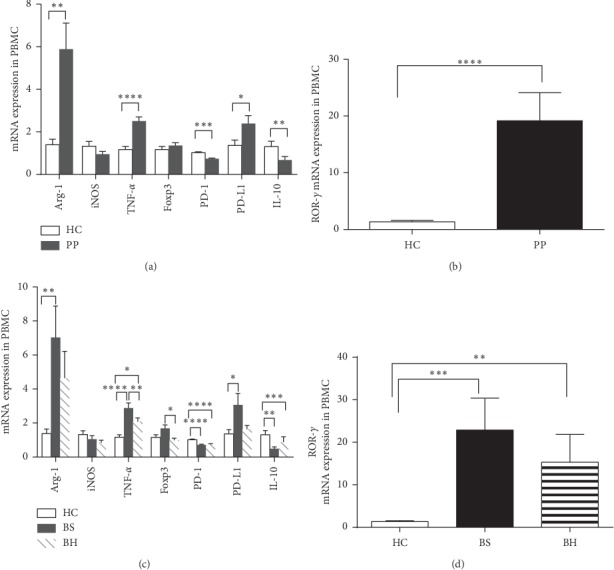
Detection of Mo-MDSC-associated immune factor mRNA in PBMCs. (a) mRNA levels of Arg-1, iNOS, IL-23, TNF-*α*, FOXP3, PD-1, PD-L1, and IL-10 in PBMCs from the HC and PP groups; (b) ROR-γ mRNA levels in PBMCs of the HC and PP groups; (c) mRNA levels of Arg-1, iNOS, TNF-*α*, Foxp3, PD-1, PD-L1, and IL-10 in PBMCs of the HC, BS, and BH groups; (d) ROR-γ mRNA levels in PBMC from the HC, BS, and BH groups (^*∗*^*p* < 0.05, ^*∗∗*^*p* < 0.01, ^*∗∗∗*^*p* < 0.001, ^*∗∗∗∗*^*p* < 0.0001).

To address the potential mechanisms of Mo-MDSC-mediated suppression in different psoriasis TCM syndrome groups and find novel laboratory biomarkers for distinguishing between the two psoriasis TCM syndrome groups, we quantified the mRNA levels of known functional mediators in psoriatic disease and Mo-MDSC-associated immune factors as well as transcription factors in the PBMCs of psoriasis patients. In our experiment, mRNA levels of Arg-1, iNOS, IL-6, IL-10, TNF-*α*, programmed cell death protein 1 (PD-1), programmed death-ligand 1 (PD-L1), IL-17A, receptor retinoic acid receptor-related orphan receptor-*γ* (ROR-*γ*), and FOXP3 were quantified by real-time RT-PCR (qRT-PCR). The detection limit of Ct values was defined as 40. Our results showed that the mRNA expression of IL-6 and IL-17A was either low or below the detection limit. Statistical analysis of qRT-PCR results indicated that the mRNA expression of Arg-1, TNF-*α*, PD-1, PD-L1, ROR-*γ,* and IL-10 in the PBMCs of psoriasis patient group was significantly different from expression levels of the control group. mRNA expression of Arg-1, TNF-*α*, PD-L1, and ROR-*γ* was increased, while the expression of PD-1 and IL-10 was decreased in the PBMCs of psoriasis patients (Figure 4). Moreover, the mRNA levels of TNF-*α* and FOXP3 in PBMCs showed a pronounced statistical difference between the BH syndrome group and the BS syndrome group.

## 4. Discussion

Psoriasis, a common chronic inflammatory skin disease, is mediated by the pathological cross-talk between epidermal keratinocytes and immune cells [19], including infiltrating CD4^+^T cells, CD8^+^T cells [20], and Mo-MDSCs [13]. Consistent with other studies [13, 21, 22], we found increased circulating CD14^+^HLA-DR^−/low^ Mo-MDSCs among CD14^+^ cells in psoriasis patients when compared to healthy controls. Further, circulating Mo-MDSCs were increased in the blood of psoriatic BS syndrome patients and not in patients with BH syndrome. These results suggested that elevated Mo-MDSCs in patients with psoriasis may result in suppression of T-cell activity. MDSCs may induce the conversion of *T* effector cells into *TA* regulatory cells in psoriasis patients [22]. Therefore, we believe that Mo-MDSCs might be beneficial for psoriatic BH syndrome patients with flared and unstable lesions.

*In vitro* data had previously shown that isolated unstimulated circulating MDSCs from psoriatic patients do not produce significant amounts of cytokines, including IL-1*β*, IL-2, IL-4, IL-5, IL-6, IL-7, IL-9, IL-10, IL-12p70, IL-13, IL-15, IL-17A, IL-17E, IL-17F, IL-21, IL-22, IL-23, TNF-*α*, and IFN-*γ* [22]. To elucidate the mechanisms of Mo-MDSC recruitment and function in psoriasis patients with BS syndrome, we analyzed MDSC-associated chronic inflammatory mediator levels in serum. In psoriatic BS syndrome patients, the levels of all immune factors measured, including IL-2, IL-4, IL-6, IL-10, IL-17A, TNF-*α*, IFN-*γ*, iNOS, Arg-1, and NO, showed no statistical difference when compared with the healthy controls and the psoriatic BH syndrome group, respectively. These results suggest that circulating Mo-MDSCs in psoriasis patients are either functionally impaired [13] or unstimulated [22]. Next, we examined molecules in pathways involved in Mo-MDSC-mediated suppression in PBMCs. We found that the mRNA expression of Arg-1, TNF-*α*, ROR-*γ*, and PD-L1 was increased, while that of PD-1 and IL-10 mRNA was decreased in the PBMCs of psoriasis patients group. This was suggestive of circulating MDSCs contributing to the recruitment of other cell types in the blood by producing cytokines, which may skew the immune response in psoriasis patients.

The immunological pathways involving IL-23 and IL-17A (IL-23/TH17 axis), as well as TNF-*α*, have been demonstrated to play pivotal roles in the pathogenesis of psoriasis [23]. IL-23, IL-17, and TNF-*α* are central mediators of psoriatic plaque formation, as shown by the fact that pharmacological blockade of either of these cytokines by monoclonal antibodies causes clinical remission [24, 25]. TNF-*α* is a potent proinflammatory cytokine secreted by a variety of immune cells in the skin, including T cells, DCs, and macrophages [26, 27]. We found that TNF-*α* mRNA expression in PBMCs was not only significantly increased in psoriatic patients when compared to the control group but also significantly increased in the psoriatic BS group when compared to the BH group. Upregulation of TNF-*α* expression is in line with the increased fraction of CD14^+^HLA-DR^−/low^ MDSC/ CD14^+^ cells seen in psoriatic BS syndrome patients, suggesting that elevated Mo-MDSCs in patients with psoriasis may be involved in the suppression of T-cell activity. Amidst the activation of proinflammatory cascades, accumulating Mo-MDSCs may exert an inhibitory effect on such proinflammation activity, potentially limiting the progression of psoriatic lesions in BS syndrome patients. The current study provides evidence that Mo-MDSCs and TNF-*α* are potential biomarkers for identifying and distinguishing between psoriatic BS syndrome and BH syndrome.

Further, the concentration of serum IL-17A in psoriasis patients was not significantly different when compared with levels in healthy controls. It is worth noting that the mRNA expression of IL-17A was low or below the detection limit in both the psoriasis patient group and the control group. IL-17 A expression is upregulated in various proinflammatory cells, which is relevant to psoriasis pathogenesis. However, only cutaneous IL-17A expression correlates with disease activity [28]. IL-17 A induces the expression of chemokines by keratinocytes, which then mediate the recruitment of Th17 cells, neutrophils, and dendritic cells into the skin [28, 29]. IL-17 A inhibitors successfully downregulate the expression of multiple chemokines, cytokines, and proteins involved in the proinflammatory response of activated Th17 cells and keratinocytes in lesioned psoriatic skin [28, 30]. Therefore, we expect that IL-17A exerted its effect at local inflammatory sites of psoriatic skin rather than the circulation. Another possibility is that Mo-MDSCs downregulate IL-17A levels in circulation [31].

The transcription factor ROR-*γ* plays a critical role in the expression of proinflammatory cytokine IL-17 in patients with psoriasis [32] and is, therefore, an attractive target for the treatment of psoriasis [33]. In the current study, we found the ROR-*γ* mRNA levels were significantly increased in the PBMCs of psoriasis patients when compared to controls. These results implicate that increased ROR-*γ* mRNA expression in the PBMCs in psoriasis patients may not mediate the Th17 immune response in peripheral blood cells. Therefore, we proposed that the upregulation of ROR-*γ* mRNA in PBMCs may drive the Th17 immune response confined to the inflamed area of the skin by inducing peripheral immune cell differentiation and localization to the inflamed skin region.

Psoriasis is known to be associated with an upregulation of Th1 and Th17 cytokines and a relative downregulation of Th2 and Treg cytokines. A Th1/Th2 and Th17/Treg imbalance is associated with psoriasis [34]. IL-10 is a cytokine produced primarily by monocytes and Th2 cells [35]. It inhibits the production of soluble proinflammatory mediators such as cytokines TNF-*α*, IL-1b, IL-6, and IL-12 or the CXC chemokine ligand 8 (CXCL8) [36]. Some authors describe a deficiency in the expression of IL-10 mRNA in psoriasis in comparison to normal skin tissue [37, 38]. We found lower IL-10 mRNA levels in PBMCs of psoriasis patients when compared to healthy controls, especially in psoriatic BS syndrome patients, in disagreement with the elevated fraction of CD14^+^HLA-DR^−/low^ MDSC/ CD14^+^ cells. Most publications have described that IL-10 levels are decreased in the patient serum [39], but some authors have also reported that IL-10 serum levels were not changed in psoriasis [40], which is in line with our data. A possible cause of our results may be that MDSCs induce a Th1/Th2 imbalance in psoriasis patients under specific conditions, such as the stationary clinical stage of psoriasis (psoriatic BS syndrome).

Treg cells maintain immune tolerance and inflammatory balance. Defects in the number and function of Treg cells can induce the development and progression of psoriasis [41]. FOXP3 is a more specific marker of Treg cells than CD4 and CD25, and it is very important for the development and activity of Treg cells [42]. In the current study, FOXP3 mRNA expression in PBMCs was not significantly different in psoriatic patients compared to healthy controls; however, it was significantly increased in the psoriatic BS syndrome group when compared to the BH syndrome group. Our evidence suggests that MDSCs may induce the conversion of *T* effector cells to *T* regulatory cells in psoriasis patients, indicating that FOXP3^+^ Treg populations are a potential biomarker for the distinction between these two psoriatic TCM syndrome groups.

PD-L1 is a key molecule that mediates the immunosuppressive activity of MDSCs via its interaction with the PD-1 receptor on T cells [43, 44]. The absolute numbers and percentages of CD4^+^PD-1^+^ and CD8^+^PD-1^+^ T cells were significantly decreased in psoriasis patients when compared to controls [45]. In this study, we found significant downregulation of PD-1 mRNA in psoriasis patient PBMCs, while PD-L1 mRNA levels showed no significant change compared to levels in PBMCs of controls. A previous study had found that Mo-MDSCs from psoriasis patients have decreased PD-1 and IL-10 expressions, while the PD-L1 expression in psoriatic MDSCs was not significantly changed when compared to healthy control Mo-MDSCs [13]. These results suggest that the deregulation of PD-1 in Mo-MDSCs, T cells, and other PBMCs may further affect the balance of immune regulation in inflamed skin regions. Interestingly, we showed that PD-L1 mRNA was upregulated in PBMCs of psoriatic BS syndrome patients compared to healthy control PBMCs. It would be important to determine the particular peripheral blood cell types in which PD-L1 mRNA is upregulated and how this change affects psoriatic BS syndrome in a future study.

This current study has a number of limitations. Firstly, only a small number of psoriasis patients, 47, were enrolled, and it was a single-center study, so the distribution of the data might not be symmetrical. Secondly, there were issues with obtaining sufficient amounts of blood for all assays that were planned, and thus there are varying numbers of repeats for some of the measurements. Finally, the techniques for the precise and accurate study of Mo-MDSC function networks in psoriatic skin and peripheral blood are limited. Future studies should employ techniques, such as cell sorting and single-cell sequencing, in order to sort Mo-MDSCs and measure associated immune cell function in psoriatic skin and peripheral blood, followed by the characterization of immune networks in different TCM syndrome groups.

Taken together, our data provides evidence that the percentage of CD14^+^HLA-DR^−/low^ MDSC/ CD14^+^ cells and TNF-*α* and FOXP3 mRNA expression levels in PBMCs are potential biomarkers for making a distinction between the psoriasis TCM BS syndrome and BH syndrome. These biomarkers might be the reason behind the difference in clinical manifestations of the two syndromes. The current results provide new insight into the immune mechanism of psoriatic TCM syndromes, which may help TCM doctors improve clinical efficacy and prepare precise prescriptions.

## Figures and Tables

**Table 1:** Primers for real-time PCR.

Target	Forward primer	Reverse
PD-1	CATTGTGGAAGGGCTCATGA	TCTTCTGGGT GGCAGTGATG
PD-L1	TGTGACCAGCACACTGAGAA	AGTCCTTTCATTTGGAGGATGT
IL-10	TGAGAACCAAGACCCAGACA	GGGAAGAAATCGATGACAGC
GAPDH	CATTGTGGAAGGGCTCATGA	TCTTCTGGGT GGCAGTGATG
IL-17	ATGACTCCTGGGAAGACCTCATTG	TTAGGCCACATGGTGGACAATCGG
TNF-*α*	GCTGCTCACCTCATTGGAG	CCAGGAGAGAATTGTTGCTCA
IL-6	CCTCTCTGCAAGAGACTTCCAT	AGTCTCCTCTCCGGACTTGT
IL-23	GTATCCAGTGTGAAGATGGTTGTGA	CGGATCCTTTGCAAGCAGAA
Foxp3	GCAGCTCTCAACGGTGGAT	GGGATTTGGGAAGGTGCAGA
iNOS	CTTTCCAAGACACACTTCACCA	TATCTCCTTTGTTACCGCTTCC
Arg1	CAAGAAGAACGGAAGAATCAGC	TTGTGGTTGTCAGTGGAGTGTT
ROR-*γ*	GTGCTGGTTAGGATGTGCCG	GTGGGAGAAGTCAAAGATGGA

**Table 2:** Patient demographics.

	Subject groups		
	Health controls	Psoriasis with BS syndrome	Psoriasis with BH syndrome
No. of patients analyzed	20	24	23
Age (y)	38 ± 12	37 ± 12	41 ± 16
Sex (n)	10 males, 10 females	15 males, 9 females	13 males, 10 females
Duration (y)	NA	13.68 ± 13.25	10.71 ± 9.71
Current treatment	None	20 none, 4 combined treatment	18 none, 5 combined treatment
Medication	None	3 oral Chinese herbs 1 topical corticosteroid	3 oral Chinese herb 1 phototherapy 1 topical calcipotriol
Psoriasis area and severity index	NA	3.35 (0.6–8) ± 2.18	3.15 (0.4–10.8) ± 2.44

Data are presented as mean (SD) and *n* (%). HC, healthy controls. NA, not applicable.

## Data Availability

Some or all data, models, or code generated or used during the study are available from the corresponding author by request.
